# Machine Learning-Based Radiomics Predicting Tumor Grades and Expression of Multiple Pathologic Biomarkers in Gliomas

**DOI:** 10.3389/fonc.2020.01676

**Published:** 2020-09-11

**Authors:** Min Gao, Siying Huang, Xuequn Pan, Xuan Liao, Ru Yang, Jun Liu

**Affiliations:** ^1^Department of Radiology, Second Xiangya Hospital, Central South University, Changsha, China; ^2^School of Computer Science and Engineering, University of New South Wales, Sydney, NSW, Australia; ^3^Lister Hill National Center for Biomedical Communications, National Library of Medicine, Bethesda, MD, United States

**Keywords:** glioma, biomarkers, machine learning, radiomics, MRI

## Abstract

**Background:**

The grading and pathologic biomarkers of glioma has important guiding significance for the individual treatment. In clinical, it is often necessary to obtain tumor samples through invasive operation for pathological diagnosis. The present study aimed to use conventional machine learning algorithms to predict the tumor grades and pathologic biomarkers on magnetic resonance imaging (MRI) data.

**Methods:**

The present study retrospectively collected a dataset of 367 glioma patients, who had pathological reports and underwent MRI scans between October 2013 and March 2019. The radiomic features were extracted from enhanced MRI images, and three frequently-used machine-learning models of LC, Support Vector Machine (SVM), and Random Forests (RF) were built for four predictive tasks: (1) glioma grades, (2) Ki67 expression level, (3) GFAP expression level, and (4) S100 expression level in gliomas. Each sub dataset was split into training and testing sets at a ratio of 4:1. The training sets were used for training and tuning models. The testing sets were used for evaluating models. According to the area under curve (AUC) and accuracy, the best classifier was chosen for each task.

**Results:**

The RF algorithm was found to be stable and consistently performed better than Logistic Regression and SVM for all the tasks. The RF classifier on glioma grades achieved a predictive performance (AUC: 0.79, accuracy: 0.81). The RF classifier also achieved a predictive performance on the Ki67 expression (AUC: 0.85, accuracy: 0.80). The AUC and accuracy score for the GFAP classifier were 0.72 and 0.81. The AUC and accuracy score for S100 expression levels are 0.60 and 0.91.

**Conclusion:**

The machine-learning based radiomics approach can provide a non-invasive method for the prediction of glioma grades and expression levels of multiple pathologic biomarkers, preoperatively, with favorable predictive accuracy and stability.

## Introduction

Gliomas are the most common brain tumors and are often classified as World Health Organization (WHO) grades I-IV, depending on the different tumor cells, and the degree of abnormality (1, 2). As a tumor’s grade increases, gliomas process more aggressively (3). Treatment options and responses differ from glioma grades (4). Pathological findings are the premise of rational treatment. Usually, glioma grades are confirmed by pathological examination during surgery or biopsy (5). Then, a following immunohistochemistry (IHC) test determines the molecular biomarkers of tumor tissues at the microscopic level. These pathologic biomarkers, typical proteins, are useful indicators for diagnosis, prognosis, or treatment response (6). However, obtaining such information for gliomas requires invasive approaches. The surgical decision making could be difficult and time-consuming for many patients. Those patients who are not eligible for a surgery or seek non-surgical treatment may have limited treatment options without pathological guidance. Therefore, presurgical glioma grades and the expression of biomarkers are valued and preferred with non-invasive approaches.

At present, the medical imaging can differentiate the tumor phenotype and intra-tumor heterogeneity (7). Conventional magnetic resonance imaging (MRI) is routinely used in the diagnosis and management of glioma patients. T1-weighted contrast-enhanced MRI (T1C) is the current standard for initial brain tumor imaging (8). Radiomics can generate image features with high dimensional data from the intensity histogram, geometry and texture analyses on the entire tumor volume (9). With the emergence of Artificial Intelligence (AI) technologies, advanced informatics tools have become accessible to facilitate machine learning (ML) based radiomics applications using image features as the data source (10). Radiomics is gaining ground in oncology and have the potential to accurately classify or predict tumor characteristics.

Radiomics approaches have been applied for the predictions of glioma grades or differential diagnoses (11, 12). Several studies have reached a prediction accuracy of above 80% using popular ML models. The commonly and frequently used ML algorithms in radiomics include Logistic Regression (LR), Random Forests (RF), Support Vector Machine (SVM), and etc. Each ML method has their own advantages in the classification. For example, LR fits the variables coefficients and predicts a logit transformation of the probability of being one class or the other. SVM separates the classes by finding an optimal hyperplane. RF uses bootstrap aggregating to decision trees and improves classification performance.

When compared to tumor grading, to make predictions at a molecular level is more challenging. Kickingereder et al. reported the association between established MRI features and cancer gene variations (EGFR amplification and CDKN2A loss), but failed to build a sufficient ML model to predict the molecular characteristics (13). In clinic, pathologic biomarkers are more frequently tested for than genetic testing. IDH1 is one important glioma biomarker and IDH1 mutation along with 1p/19q is a part of the molecular diagnosis in the updated 2016 WHO classification (14). Ki67, S100, and GFAP are also the common protein targets for gliomas. IDH1, Ki67, and GFAP were once considered as the golden triad of glioma IHC (15) Ki67 is highly correlated to proliferation that may indicate the tumor grades and prognosis (16–18). S100 has been implicated in the regulation of cellular activities, such as metabolism, motility, and proliferation. Under the pathological conditions of tumor and inflammation, the concentration of the S100 protein increases to the micromole level, which stimulates microglia and astrocytes, and increases the expression of pro-inflammatory cytokines (19–23). GFAP is the most widely used markers of astrocytes (24). Under the condition of injury (trauma or disease), the expression of GFAP in astrocytes rapidly increases (25). GFAP is often used to reveal the astrocytic lineage of glial cells and glial tumor cells, and plays a more significant role in tumor pathology, when compared to the differential diagnosis of astrocytoma. Ki67, S100, or GFAP may not be a reliable diagnostic biomarker for gliomas, because their roles in gliomas are still under investigations, while controversies have been observed in experiments (26). However, there is no doubt that these proteins can provide some insights into the tumor intra-microenvironment.

So far, it is not surprising to know that most radiomics studies favor the prediction of the IDH expression for molecular diagnosis (11, 27), with a few reports on Ki67 (28). In order to expand predictive effects of radiomics, the investigators aimed to assess the prediction feasibility of glioma grades and the pathologic biomarkers of Ki67, S100, and GFAP in gliomas. The investigators believed that the combination of multiple biomarkers can increase the predictive power, and the information obtained can help in understanding the underlying pathologic process in gliomas. The investigators designed the present retrospective study and extracted hundreds of radiomic features from the T1C images of 367 glioma patients. Three machine-learning-based models (LR, SVM, and RF) were built to perform the tasks: (1) classify the glioma grades, and (2) predict the expression levels of Ki67, S100, and GFAP. This study demonstrated that multiple pathologic biomarkers in gliomas can be estimated to the certainty levels of clinical using common ML models on conventional MRI data and pathological records.

## Materials and Methods

### Study Cohort

The investigators retrospectively collected a data set of 420 glioma patients, who had pathological reports and MRI scans performed between October 2013 and March 2019, from the Second Xiangya Hospital of Central South University. The patients who met the following criteria were included: (i) a histopathological diagnosis of primary glioma based on the WHO classification, (ii) the availability of IHC profiles of biomarkers (S100, GFAP, and Ki67), (iii) preoperative MRI data of post-contrast axial T1-weighted (T1C), and (iv) age > 18 years old. Patients were excluded due to the following: (i) secondary gliomas or postoperative recurrence of gliomas, (ii) obvious artifacts in MRI. Ethics approval was obtained for the present study from the Ethics Committee of the Second Xiangya Hospital, Central South University.

### Pathological Evaluation

Patient demographics (age and gender), and histopathologic diagnosis and IHC results were obtained from a surgical pathology report. On these reports, the diagnosis included a specific glioma type by cells (e.g., astrocytoma and oligodendrogliomas) and a given WHO grade (I–IV). The IHC results were presented in the list of glioma biomarkers (e.g., S100, GFAP, or Ki67) and their own expression profile in tumor cells. It is noteworthy that the list was not standard and varied upon the request or availability of the biomarkers at that time. For example, few patients received an IDH1 test before 2017, but after 2016, the WHO classification standard was published, and IDH1 tests became common. So, a patient might have a different set of tested biomarkers, and the number of cases can differ for each biomarker. Their IHC results depended on the scoring system used. The expression levels were usually evaluated by the staining intensity of positive cells, and points were assigned to describe these positive cells by count (e.g., 0 points as negative (−), 1 point as positive (+), 2 points as medium positive, and 3 points as high positive), percentage (e.g., 0 points as none, 1 point less than 5%, 2 points approximately 5–25%, and 3 points above 25%), or the appearance of a clear brown color (e.g., 1 point for light yellow). In the study, the glioma grades were classified as low-grade (WHO I–II, benign) and high-grade (WHO III–IV, malignant), and expression levels of biomarkers were divided into two categories: a low expression scored less than 2 points and a high expression scored 2 points or above.

### Imaging Post-processing and Radiomics Features Extraction

Magnetic resonance imaging scans were acquired from different scanners over time. The Picture Archiving and Communication System (PACS) exported the selected DICOM images to a local computer using the RadiAnt DICOM Viewer (Medixant, PL). In order to reduce the influence of different scanning parameters, post-processing and image registration were applied using the Advanced Normalization Tools (ANTS 2.1, PA). Then, the DICOM images were loaded into ITK-SNAP for segmentation and standardization (29). Two neuroradiologists (5 years of experience) drew the region of interest (ROI) around the tumor boundary on the T1C images. The neuroradiologists were blinded to the patient identification and diagnosis. After a joint effort, disagreements with the boundary were solved. The ROI segmentations were resampled to match the dimensions of the original images, and both images were saved in.narrd as the input for feature extraction.

The Pyradiomics extractor was customized to calculate and extract the features (10). All built-in filters [wavelet, Laplacian of Gaussian (LoG), square, square root, logarithm, and exponential] were enabled on five image feature classes [first order statistics, shape descriptors, and texture features on the gray-level co-occurrence matrix (GLCM), gray-level run length matrix (GLRLM), and gray-level size zone matrix (GLSZM)]. Feature definitions and calculation algorithms were available in the PyRadiomics documentation^1^.

### Machine Learning

The feature importance and the following predictive ML methods were implemented using Python (version 3.7.0) with machine-learning library scikit-learn (version 23.0) (30). All features were standardized through Min-Max scaling. Features with all zero scores were removed. Clinical data (age and gender) were added in constructing the final prediction models.

#### Feature Importance

The feature importance helped in understanding the importance of the features, since a large number radiomics features with high-dimensional data are difficult to interpret. Three technique approaches were used to identify the important features. First, chi-squared (chi^2^) tests were applied in the scikit-learn SelectKBest class to obtain a list of the top 15 best features. Second, the heatmap of correlated features was plotted to identify features highly correlated to predicting targets (glioma grade and biomarker expression) using the seaborn library. Third, a RF classifier was initiated and the in-build feature importance was used to extract the top features.

#### Predictive Machine Learning Models

Three frequently-used machine-learning based models of LR, SVM, and RF were built for four predictive tasks: (1) glioma grades, (2) Ki67 expression level, (3) GFAP expression level, and (4) S100 expression level in gliomas. Each sub dataset was divided into training and testing sets at a ratio of 4:1 (train_size = 0.8, test_size = 0.2). Principal Component Analysis (PCA) was applied for high-dimension reduction that maps *n*-dimensional features to *k*-dimensional features (*n* > *k*), resulting in brand new orthogonal features. For the unbalanced data in different classes, the synthetic minority over-sampling technique (SMOTE) algorithm was used to oversample the minority class (31). On training set, the grid search with cross-validation was applied for hyper parameters tuning (RF and SVM), and *k* fold validation was used for LR. The accuracy score was compared with the result from their base models (default settings in scikit-learn) for model selection. The testing set was used for final model evaluation. The performance of the models was evaluated according to accuracy, the area under curve (AUC) of the receiver operating characteristic (ROC), sensitivity, specificity, the positive prediction value (PPV), and the negative predictive value (NPV). According to the AUC and accuracy, the best classifier was chosen for each task.

### Statistics

One way-ANOVA or simple *t*-test was applied to test the differences among gender, age, glioma grade, and the expression levels of the biomarkers. Descriptive statistics was used to summarize the important features through filters and feature classes. All significant levels were tested at 0.05.

## Results

### Subjects and Pathologic Biomarkers

A data set of preoperative MRI and surgical pathologic reports of 420 glioma patients were collected. A total of 51 patients were excluded for not meeting the inclusion criteria. Among these patients, 40 patients were under 18 years old, seven patients had quality issues on their MRI data, and four patients did not have an assigned WHO classification level in their records. The age of the enrolled 369 patients ranged within 18–75 years old (mean age: 45.63 ± 13.22 years old), and consisted of 210 males (age: 46.99 ± 13.24 years old), and 159 females (age: 43.84 ± 13.03 years old). The clinical characteristics of patients and the distribution of the selected biomarkers across glioma grades are presented in Table 1.

**TABLE 1:** Distribution of clinical characteristics and expression levels of IHC biomarkers grouped by glioma WHO grades.

	**WHO I**	**WHO II**	**WHO III**	**WHO IV**
Total number	5	142	116	106
Mean age (s.d.)	35.4(7.64)	40.65(11.69)	48.29(13.82)	49.87(12.4)
Gender				
*Male*	2	72	68	68
*Female*	3	70	48	38
Tumor volume av a (cm^3^)	30.8	38.34	46.47	53.81
Ki67 expression level				
0	4	73	15	4
1	0	57	97	98
GFAP expression level				
0	0	1	1	2
1	5	126	98	94
2	0	13	13	9
3	0	2	2	1
S100 expression level				
0	0	3	0	5
1	5	120	104	86
2	0	6	5	4

The expression of GFAP, Ki67, and S100 was reported as follows: 367 patients had GFAP results with four negatives (0 point), 323 positives (1 point), and 35 medium (2 points), or 5 high positives (3 points); 348 patients underwent Ki67 tests, including 96 negatives or low positives (≤5% in tumor cells), and 252 strong positives (>5%); 338 patients underwent S100 tests, which included eight negatives (0 points), 315 positives (1 point), and 15 medium positives (2 points).

There was a significant age difference among male and female patients, as determined by one-way ANOVA [*F* (1, 367) = 5.17, *P* < 0.05]. Furthermore, there were significant differences in age, gender and tumor volume among glioma grades (WHO I–IV). Moreover, there were significant differences in glioma grade, tumor size, age and gender for the Ki67 expression. However, there were no significant differences in age, gender and glioma grade for S100 and GFAP expression. The *t*-test and one-way ANOVA results are shown in Table 2.

**TABLE 2:** Clinical characteristics vs. glioma grade and expression levels of IHC biomarkers.

	**Age**	**Gender**	**Tumor volume**	**Grade**
Grade	*t* = 6.1602 df = 367 *p* = 1.91e-09	*t* = −2.2766 df = 367 *p* = 0.02339	*t* = 2.5027 df = 355 *p* = 0.01277	
Ki67	*t* = 5.6168 df = 346 *p* = 4.001e-08	*F*(1,346) = 0.53 *p* = 0.467	*t* = 1.5089 df = 336 *p* = 0.1323	*F*(1,346) = 124.7 *p* < 0.05
GFAP	*t* = −0.30242 df = 365 *p* = 0.7625	*F*(1,365) = 0.569 *p* = 0.451	*t* = −1.1268 df = 354 *p* = 0.2606	*F*(1,365) = 0.089 *p* = 0.77
S100	*t* = −0.307 df = 336 *p* = 0.759	*F*(1,336) = 0.186 *p* = 0.667	*t* = 1.639 df = 326 *p* = 0.1022	*F*(1,336) = 0.59 *p* = 0.44

### MRI Data Processing and Feature Extraction

A total of 369 original T1C images and their paired segmentation images underwent the feature extraction process using Pyradiomics. The investigators extracted 1,421 radiomics features (14 shape features, 27 first-order intensity statistics features, 68 texture features, 96 square features, 96 square root features, 96 logarithm features, 96 exponential features, 172 LoG features, and 766 wavelet features). After data cleaning, 1,372 features reminded. The data set was normalized by the SKlearn MinMaxScaler.

### Features Importance

**FIGURE 1:**
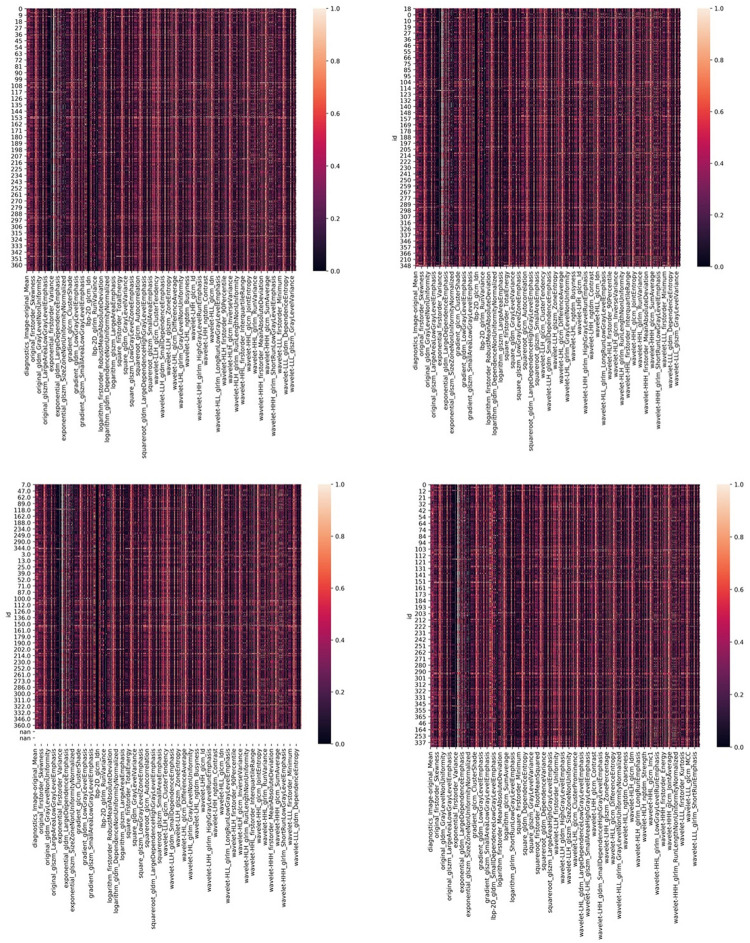
The heatmaps of corelated features for glioma grade and biomarkers of Ki67, GFAP, and S100.

**FIGURE 2:**
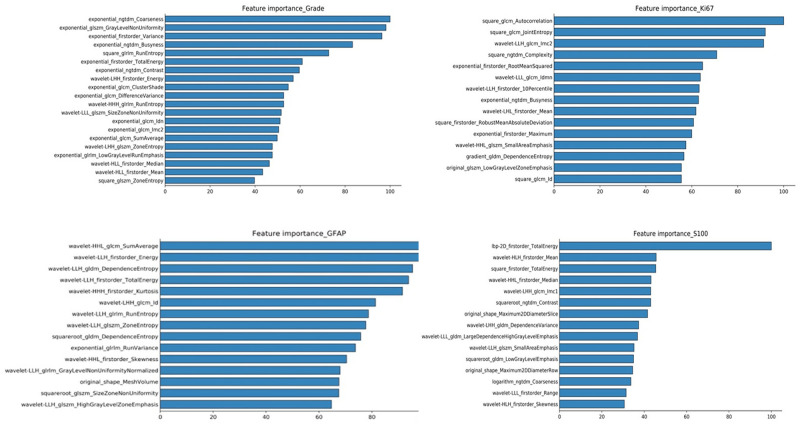
RF model inbuild feature importance for predicting glioma grades and biomarkers of Ki67, GFAP, and S100.

The investigators obtained the list of the top 15 important features based on the scores obtained from the chi-squared stats between each non-negative feature and the glioma grade, and S100, GFAP, and Ki 67 expression levels. The features and their scores are shown in Table 3. The scores ranged within 3.67–44.04. The mean score of the top important features was 9.30, with a standard deviation of 5.83. The frequent top features within the image type were exponential (23), wavelet (22), square (6), square root (3), original (3), gradian (2), and ihp-2D (1). For the feature classes, the frequent top features were divided as follows: glszm (27), glcm (9), glrlm (8), gldm (7), first order (7), and ngtdm (2). The heatmaps of the correlated features for glioma grade and the biomarkers of Ki67, GFAP, and S100 are presented in Figure 1. The RF model built-in feature importance is presented in Figure 2.

**TABLE 3:** Feature importance by chi-square scores.

**Prediction**	**Top pyradiomics imaging feature**	**Score**	**Filter**	**Class**
GRADE	Exponential_ngtdm_Coarseness	44.04	Exponential	ngtdm
	Exponential_glszm_LowGrayLevelZoneEmphasis	19.79	Exponential	glszm
	Exponential_glszm_SizeZoneNonUniformityNormalized	16.93	Exponential	glszm
	Exponential_glszm_ZoneEntropy	13.68	Exponential	glszm
	Exponential_glcm_MCC	12.09	Exponential	glcm
	Exponential_glcm_Correlation	11.30	Exponential	glcm
	Exponential_glszm_GrayLevelNonUniformity	10.92	Exponential	glszm
	Exponential_glszm_SmallAreaEmphasis	10.60	Exponential	glszm
	Exponential_glcm_InverseVariance	10.52	Exponential	glcm
	Square_glszm_ZonePercentage	9.80	Square	glszm
	Wavelet-LHL_firstorder_TotalEnergy	9.64	Wavelet-LHL	firstorder
	Exponential_glcm_Imc2	9.59	Exponential	glcm
	Exponential_glszm_GrayLevelNonUniformityNormalized	9.46	Exponential	glszm
	Gradient_firstorder_TotalEnergy	9.03	Gradient	firstorder
	Wavelet-HHL_firstorder_TotalEnergy	8.48	Wavelet-HHL	firstorder
GFAP	Lbp-2D_firstorder_10Percentile	12.38	Lbp-2D	firstorder
	Wavelet-HLH_glrlm_LowGrayLevelRunEmphasis	12.25	Wavelet-HLH	glrlm
	Wavelet-HLH_gldm_LowGrayLevelEmphasis	12.13	Wavelet-HLH	gldm
	Wavelet-HLH_glszm_LowGrayLevelZoneEmphasis	11.79	Wavelet-HLH	glszm
	Wavelet-HHH_gldm_LowGrayLevelEmphasis	11.19	Wavelet-HHH	gldm
	Wavelet-HHH_glrlm_LowGrayLevelRunEmphasis	11.18	Wavelet-HHH	glrlm
	Wavelet-HHL_gldm_LowGrayLevelEmphasis	11.12	Wavelet-HHL	gldm
	Wavelet-HHL_glrlm_LowGrayLevelRunEmphasis	11.08	Wavelet-HHL	glrlm
	Wavelet-HLH_gldm_LargeDependenceLowGrayLevelEmphasis	10.77	Wavelet-HLH	gldm
	Wavelet-HHH_gldm_LargeDependenceLowGrayLevelEmphasis	10.64	Wavelet-HHH	gldm
	Wavelet-HHH_glrlm_LongRunLowGrayLevelEmphasis	9.99	Wavelet-HHH	glrlm
	Wavelet-HHL_gldm_LargeDependenceLowGrayLevelEmphasis	9.89	Wavelet-HHL	gldm
	Wavelet-HLH_glrlm_ShortRunLowGrayLevelEmphasis	9.30	Wavelet-HLH	glrlm
	Wavelet-HHL_glrlm_LongRunLowGrayLevelEmphasis	8.98	Wavelet-HHL	glrlm
	Wavelet-HHH_glrlm_ShortRunLowGrayLevelEmphasis	8.51	Wavelet-HHH	glrlm
S100	Wavelet-LLH_glszm_LargeAreaHighGrayLevelEmphasis	13.65	Wavelet-LLH	glszm
	Wavelet-LLL_glszm_LargeAreaHighGrayLevelEmphasis	10.53	Wavelet-LLL	glszm
	Original_glszm_LargeAreaHighGrayLevelEmphasis	10.45	Original	glszm
	Squareroot_glszm_LargeAreaHighGrayLevelEmphasis	8.44	Squareroot	glszm
	Original_glszm_ZoneVariance	8.08	Original	glszm
	Exponential_firstorder_Energy	7.89	Exponential	firstorder
	Original_glszm_LargeAreaEmphasis	7.87	Original	glszm
	Squareroot_glszm_ZoneVariance	5.93	Squareroot	glszm
	Squareroot_glszm_LargeAreaEmphasis	5.83	Squareroot	glszm
	Exponential_firstorder_TotalEnergy	5.72	Exponential	firstorder
	Wavelet-LHH_glszm_LargeAreaLowGrayLevelEmphasis	5.65	Wavelet-LHH	glszm
	Wavelet-LLH_glszm_ZoneVariance	5.49	Wavelet-LLH	glszm
	Wavelet-LLH_glszm_LargeAreaEmphasis	5.39	Wavelet-LLH	glszm
	Gradient_glszm_LargeAreaLowGrayLevelEmphasis	5.23	Gradient	glszm
	Wavelet-LHL_glszm_LargeAreaHighGrayLevelEmphasis	4.98	Wavelet-LHL	glszm
Ki67	Exponential_ngtdm_Coarseness	18.37	Exponential	ngtdm
	Exponential_glszm_LowGrayLevelZoneEmphasis	8.44	Exponential	glszm
	Exponential_glszm_SizeZoneNonUniformityNormalized	7.75	Exponential	glszm
	Exponential_glszm_ZoneEntropy	6.12	Exponential	glszm
	Exponential_glszm_GrayLevelNonUniformity	4.64	Exponential	glszm
	Exponential_glcm_MCC	4.36	Exponential	glcm
	Square_glszm_SmallAreaLowGrayLevelEmphasis	4.20	Square	glszm
	Square_gldm_LowGrayLevelEmphasis	4.12	Square	gldm
	Exponential_glcm_Imc2	4.12	Exponential	glcm
	Square_glrlm_LowGrayLevelRunEmphasis	4.09	Square	glrlm
	Exponential_glcm_InverseVariance	4.02	Exponential	glcm
	Exponential_glszm_GrayLevelNonUniformityNormalized	3.95	Exponential	glszm
	Exponential_glcm_Correlation	3.89	Exponential	glcm
	Square_firstorder_Uniformity	3.69	Square	firstorder
	Square_glcm_MaximumProbability	3.67	Square	glcm

### Prediction Machine Learning Models

**FIGURE 3:**
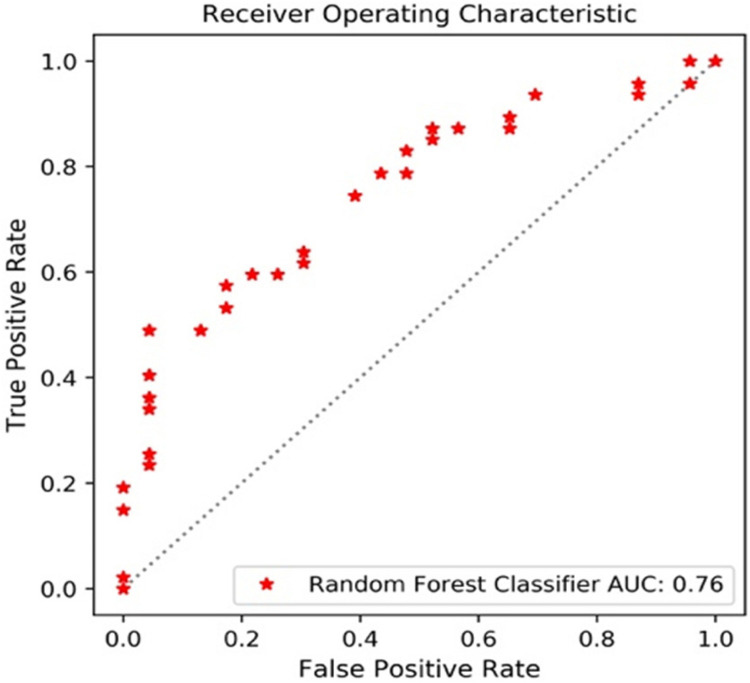
AUC_ROC for the RF classifier.

The performance of the 12 predictive models is presented in Table 4. The RF models performed slightly better, when compared to the other models. The comparisons with accuracy and the results are presented below. Figure 3 shows the AUC_ROC for the RF classifier in sub test sets.

**TABLE 4:** The performance of predictive models.

**Models**	**Error rate**	**True positive rate**	**True negative rate**	**AUC**	**Score (mean accuracy)**
Logistic_Ki67	0.22857	0.787234	0.73913	0.799	0.771429
SVM_Ki67	0.25714	0.851064	0.521739	0.748	0.742857
Random Forest_Ki67	0.2	0.914894	0.565217	0.849	0.8
Logistic_GFAP	0.24324	0.615385	0.786885	0.774	0.756757
SVM_GFAP	0.21622	0.153846	0.918033	0.613	0.783784
Random Forest_GFAP	0.18919	0.076923	0.967213	0.718	0.810811
Logistic_S100	0.19118	0	0.859375	0.164	0.808824
SVM_S100	0.11765	0	0.9375	0.48	0.882353
Random Forest_S100	0.08824	0	0.96875	0.604	0.911765

#### Glioma Grades

The sub data set was randomly split into the training set of 276 cases and the test set of 93 cases. With a PCA retention of 0.95, the PCA process reduced the dimensions to 37 components, and these remained in the final prediction model of glioma grading. There was a 96:252 class distribution. After SMOTE oversampling, the number of train samples increased to 318. After grid search with cross validation (cv = 5) or K fold validation (n_splits = 5), the selected classifier included: (1) LR (penalty = “l2,” C = 1.0), (2) SVM (C = 10, kernel = “rbf,” and gamma = 0.1), and (3) RF (min_samples_leaf = 1,min_samples_split = 2, and n_estimators = 100). The RF classifier achieved a satisfying predictive performance (AUC: 0.79, accuracy: 0.81). The average accuracy, sensitivity, specificity and f1 score was 0.81, 0.63, 0.89, and 0.67, respectively.

#### Ki67 Expression

A total of 348 patients had Ki67 test results, which included 252 low expression levels and 96 high expression levels. There was a 96:252 class distribution. The training set and test set were split into 278 and 70 cases, respectively. After the SMOTE oversampling, the number of train samples increased to 415. With a PCA retention of 0.95, the PCA process reduced the dimensions to 37 components, and there were used for the final prediction model for the Ki_67 expression. After grid search with cross validation (cv = 5) or K fold validation (n_splits = 5), the selected classifier included: (1) LR (penalty = “l2,” C = 1.0), (2) SVM (C = 10, kernel = “rbf,” and gamma = 0.1), and (3) RF (max_depth = 80, max_features = 3, min_samples_leaf = 4,min_samples_split = 8, and n_estimators = 100). Among these three classifiers, the RF classifier achieved the best predictive performance on the Ki67 expression based on the AUC (0.85), accuracy (0.80), sensitivity (0.91), specificity (0.80), and f1 score (0.85) for the Ki67 high expression.

#### S100 Expression

A total of 338 patients had S100 test results, which included 323 low expression levels (<2 points) and 15 high expression levels (≥2 points). The class distribution was 323:15. The training set and test set were split into 270 and 68, respectively. After the SMOTE oversampling, the resampled number increased to 518. With a PCA retention of 0.95, the PCA process reduced the dimensions to 38 components, and these were used for the final prediction model for the S100 expression. After grid search with cross validation (cv = 5) or K fold validation (n_splits = 5), the selected classifier included: (1) LR (penalty = “l2,” C = 1.0), (2) SVM (C = 1, kernel = “rbf,” and gamma = “auto”), and (3) RF (min_samples_leaf = 1,min_samples_split = 2, and n_estimators = 100). Among these classifiers, the RF classifier achieved the best prediction performance on the S100 expression, based on the measurements (AUC: 0.60, accuracy: 0.91, average-weighted sensitivity: 0.88 specificity: 0.91, and f1 score: 0.90). It is noteworthy that the average-weight computes f1 for each class, and returns the average while considering the proportion for each class in the dataset. For S100 low expression levels: accuracy (0.95), sensitivity (0.94), specificity (0.97), and f1 (0.95). For high expression levels: none of the four high expression cases was correctly predicted.

#### GFAP Expression

A total of 367 patients had a GFAP test. Among these patients, there were 327 low expression levels and 40 high expression levels. The class distribution ratio was 327:40. The training set and test set were split into 293 and 74, respectively. After the SMOTE oversampling, the number of samples increased to 532. With a PCA retention of 0.95, the PCA process reduced the dimensions to 38 components, and those that remained were used for the final prediction model for the GFAP expression. After grid search with cross validation (cv = 5) or K fold validation (n_splits = 5), the selected classifier included: (1) LR (penalty = “l2,” C = 1.0), (2) SVM (C = 1, kernel = “rbf,” and gamma = “auto”), and (3) RF (min_samples_leaf = 1,min_samples_split = 2, and n_estimators = 100). Among these three classifiers, the RF classifier achieved the best predictive performance on the GFAP expression measured, as follows: AUC (0.72), accuracy (0.81), average-weighted sensitivity (0.74), specificity (0.81), and f1 score (0.76).

## Discussion

The machine-learning based radiomics approach was applied to predict glioma grades and the expression levels of pathologic biomarkers Ki67, GFAP, and S100 in low or high. The overall performance of the ML models was satisfactory. The RF algorithm was found to be stable and consistently performed better than LR and SVM. Feature importance varies on predictive tasks, glioma grade or specific protein expression. The most frequent important feature classes were textual and first order statistics.

We selected LR, SVM, and RF as classifiers mainly for their popularity. LR, SVM, and RF classifiers can work on non-text data set less than 100K. Whether the data is linearly divisible or not, the linearly separable models (LR, SVM), and the non-linear separable model (RF) are helpful to view the effect and avoid the impact due to poor data. LR shows a higher AUC, in GFAP’s prediction model, but performs worst in S100’s prediction. Comparing the overall results from three biomarker prediction models, the combination of PCA reduction and RF classification consistently performed best. It suggests a common ML pipeline that may be helpful in standardizing the prediction process of multiple protein expressions.

Also more recently, researchers have demonstrated achievements of deep learning (DL) in the image segmentation and glioma grades prediction (32–37). Convolutional neural networks (CNNs) started outperforming other methods on several high-profile image analysis projects. DL has advantages in computation, as high-performance graphics processing unit (GPU) supports fast computing and less time on modeling. Like a kind of end-to-end learning, DL can automatically extract relevant functions from images, and tasks such as raw data processing and classification can be completed automatically. However, DL is complex and requires thousands of images to start with, otherwise due to a relatively small collection of images like ours, overfitting is more likely. The classic ML methods met our needs and suited the data. RF models performed well for predicting glioma grades and pathologic biomarkers S100, Ki67, and GFAP.

As it is known, the roles of these biomarkers can be complicated and controversial in laboratory experiments (26). In addition to the abilities of predicting tumor phenotypes, radiomics might offer a new approach to evaluate biomarkers, since their differentiation can be identified through the analysis of imaging features. The expression level of Ki67 was significantly correlated with the tumor grade and tumor volume, as well as the patient age and gender. A study once reported that the high level of Ki-67 expression was correlated to poor overall survival (OS) and progression free survival (PFS) (16). The accurate prediction of high level Ki67 is more meaningful than its low level expression to indicate poor prognosis for glioma patients.

The GFAP has been widely expressed in gliomas. Merely four patients presented as GFAP negative. The majority of the patients (323 of 367, 88%) had GFAP positive (+), and 327 patients with low expression GFAP (90%), combined with four that scored (−), were distributed all over the gliomas grades, including low grade (132, 40%), and high grade (195, 60%). The minority of the patients (40 of 367, 12%) had GFAP medium positive (++) or high positive (+++) distributed in low grade (15, 37.5%) and high grade (25, 62.5%). In the literature, a high GFAP expression is likely to be found in low grade gliomas. The present result was confusing, that is, the high and low expression levels of GFAP were more correlated to high grade gliomas. This result may echo that GFAP is not a direct predictor of low grade gliomas (15, 26). On the classification report of the RF_GFAP model, the accuracy score of predicting a GFAP low expression was up to, while that of predicting high expression levels of GFAP was much lower. The overall prediction performance might not be meaningful, since GFAP was lowly expressed in 90% of patients, and the model could always answer 90% correctly. The same problem was found in the predictive model of S100. It required the rethinking of these two models. There was a need to determine which expression class is more valued. And then, as one solution, the ROC thresholds can tuned, increasing the sensitivity of the favored class.

**FIGURE 4:**
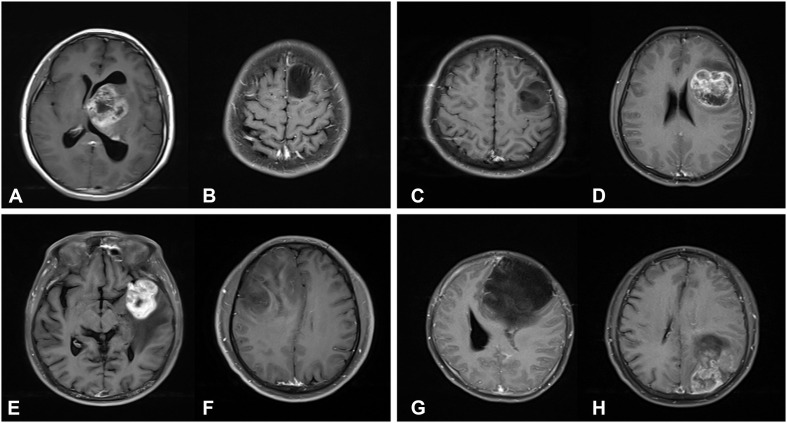
T1-weighted contrast-enhanced MR images. **(A)** A 23-year-old female patient with a grade IV glioma in left thalamus. The expression of S100β is strongly positive (S100β+++). **(B)** A 23-year-old male patient with a grade II glioma in left frontal lobe. The expression of S100β is weakly positive (S100β+). **(C)** A 27-year-old male patient with a grade II glioma in left frontal lobe. The expression of GFAP is strongly positive (GFAP+++). **(D)** A 27-year-old female patient with a grade IV glioma in left frontotemporal lobe. The expression of GFAP is weakly positive (GFAP+). **(E)** A 64-year-old male patient with a grade IV glioma in left frontotemporal lobe. The Ki67 index is 80%. **(F)** A 44-year-old male patient with a grade II glioma in right frontal lobe. The Ki67 index is 80%. **(G)** A 31-year-old female patient with a grade II glioma in left frontal lobe. Genetic test showed that IDH1 was mutant type. **(H)** A 50-year-old male patient with a grade IV glioma in left parietal-occipital lobe. Genetic test showed that IDH1 was wild type.

The interpretation of the predicted results is complex, but may be helpful to understand the molecular mechanisms it underlies. In addition, the investigators selected CE MRI from several typical cases for demonstration, in which the different expression levels of biomarkers exhibited different imaging characteristics (Figure 4). For the high expression of S100 case (Figure 4A), the tumor exhibited an obvious rosette enhancement, no enhancement of internal necrotic components, and a few edema zones around it, and was diagnosed as glioblastoma (WHO IV grade). In the image of the tumor with a low expression of S100 (Figure 4B), the tumor mass effect was obvious, but there was no obvious enhancement, and the surrounding edema was not obvious, which was diagnosed as astrocytoma (WHO II grade). In this case, the positive correlation appeared as both the S100 and glioma grade moved in the same direction that was contrary to many observations. The study conducted by Wang et al. has proven that S100 is expressed in most gliomas, and that this is an important inducer of CCL2 (19). CCL2 participates in the transport of tumor-associated macrophages (TAM) in gliomas, which affects angiogenesis, invasion, local tumor recurrence and immunosuppression. This may explain the relationship between the degree of tumor enhancement and the expression of S100 in the present cases.

There are some limitations in our study. First, we only used conventional MRI sequences with a default set of tumor features extracted by Pyradiomics. Advanced MRI sequences (e.g., DWI, DKI, MRS, ASL, et al.) can reflect the microstructure and metabolic information of tumors. In future study, we will further investigate the molecular phenotype of gliomas using a multimode magnetic resonance scheme. Second, we only selected 3 common pathologic biomarkers for gliomas from a wide range of biomarkers either current available or under investigation. We have to develop an evaluation plan for other glioma biomarkers and find candidates that can be benefit from radiomics applications. Third, imbalance classes did not reflect the incidences of glioma in real world, where glioblastoma is the most common subtype, and grade I glioma is relatively rare in adults. We used the SMOTE algorithm to balance data, oversampling the minority class, but the differences in data distribution cannot be ignored. In our experiments, before and after the use of SMOTE, AUC was only changed slightly. A larger dataset from multiple sites is expected to complement predictive effects, and the resulting classifiers can be more accurate and stable. Fourth, after PCA reducing feature dimensions, a new set of features was less remained but difficult to interpret. A combination of hierarchical clustering on PCA may help us to select feature more efficiently. At the current stage, a real-world application is out of our scope, but further prospective assessment is warranted. Based on the results we obtained as a reference, we will extend the study to identify the best classifier algorithm and the best set of features to simplify the classification tasks. The standardized computation methods would greatly enhance the reproducibility of radiomics studies, and it may also lead to standardized software solutions available in clinical practice.

In conclusion, the machine-learning based radiomics application provided a non-invasive approach for the prediction of glioma grades and expression levels of multiple pathologic biomarkers, with favorable predictive accuracy and stability. The study also demonstrated the potential of radiomics for pathological assessment and individualized cancer treatment.

## Data Availability Statement

The raw data supporting the conclusions of this article will be made available by the authors, without undue reservation.

## Ethics Statement

The studies involving human participants were reviewed and approved by Ethics committee of the second Xiangya hospital of central south university. Written informed consent for participation was not required for this study in accordance with the national legislation and the institutional requirements.

## Author Contributions

JL, MG, and SH: conception and design, and provision of study materials or patients. JL and RY: administrative support. MG, SH, XP, XL, and JL: collection and assembly of data. MG, SH, XP, and JL: data analysis and interpretation. All authors: writing and final approval of the manuscript.

## Conflict of Interest

The authors declare that the research was conducted in the absence of any commercial or financial relationships that could be construed as a potential conflict of interest.

## Footnotes

1 https://pyradiomics.readthedocs.io/en/latest/features.html

## References

[1] Louis D Ohgaki H Wiestler O Cavenee W Burger P Jouvet A The 2007 WHO classification of tumours of the central nervous system. *Acta Neuropatholo.* (2007) 114:97–109. 10.1007/978-94-007-1399-4_10 PMC1929165 17618441

[2] Omuro A DeAngelis L. Glioblastoma and other malignant gliomas: a clinical review. *JAMA.* (2013) 310:1842–50. 10.1001/jama.2013.280319 24193082

[3] Ostrom QT Gittleman H Farah P Ondracek A Chen Y Wolinsky Y CBTRUS statistical report: primary brain and central nervous system tumors diagnosed in the United States in 2006-2010. *Neuro Oncol.* (2013) 15(Suppl. 2):1–56. 10.1093/neuonc/not151 24137015 PMC3798196

[4] James M Rafay A Matthew O Frank L Misun H. Malignant gliomas: current perspectives in diagnosis, treatment, and early response assessment using advanced quantitative imaging methods. *Cancer Manag Res.* (2014) 6:149–70. 10.2147/cmar.s54726 24711712 PMC3969256

[5] Jackson R Fuller G Abi-Said D Lang F Gokaslan Z Shi W Limitations of stereotactic biopsy in the initial management of gliomas. *Neuro Oncol.* (2001) 3:193–200. 10.1215/15228517-3-3-193 11465400 PMC1920616

[6] Kristensen BW Priesterbach-Ackley LP Petersen JK Wesseling P. Molecular pathology of tumors of the central nervous system. *Ann Oncol.* (2019) 30:1265–78. 10.1093/annonc/mdz164 31124566 PMC6683853

[7] García-Figueiras R Baleato-González S Padhani A Luna-Alcalá A Vallejo-Casas J Sala E How clinical imaging can assess cancer biology. *Insights Into Imaging.* (2019) 10:28. 10.1186/s13244-019-0703-0 30830470 PMC6399375

[8] Villanueva-Meyer JE Mabray MC Soonmee C. Current clinical brain tumor imaging. *Neurosurgery.* (2017) 81:3 10.1093/neuros/nyx103 PMC5581219 28486641

[9] Chaddad A Kucharczyk M Daniel P Sabri S Jean-Claude B Niazi T Radiomics in glioblastoma: current status and challenges facing clinical implementation. *Front Oncol.* (2019) 9:374. 10.3389/fonc.2019.00374 31165039 PMC6536622

[10] Griethuysen JJMV Fedorov A Parmar C Hosny A Aerts HJWL. Computational radiomics system to decode the radiographic phenotype. *Cancer Res.* (2017) 77:e104–7. 10.1158/0008-5472.can-17-0339 29092951 PMC5672828

[11] Zhang B Chang K Ramkissoon S Tanguturi S Bi WL Reardon DA Multimodal MRI features predict isocitrate dehydrogenase genotype in high-grade gliomas. *Neuro Oncol.* (2016) 19:109–17. 10.1093/neuonc/now121 27353503 PMC5193019

[12] Lu CF Hsu FT Hsieh LC Kao YCJ Cheng SJ Hsu BK Machine learning-based radiomics for molecular subtyping of gliomas. *Clin Cancer Res.* (2018) 24:4429–36. 10.1158/1078-0432.ccr-17-3445 29789422

[13] Kickingereder P Bonekamp D Nowosielski M Kratz A Sill M Burth S Radiogenomics of glioblastoma: machine learning–based classification of molecular characteristics by using multiparametric and multiregional MR imaging features. *Radiology.* (2016) 2016:161382. 10.1148/radiol.2016161382 27636026

[14] Louis DN Perry A Reifenberger G Von Deimling A Figarella-Branger D Cavenee WK The 2016 World Health Organization classification of tumors of the central nervous system: a summary. *Acta Neuropathol.* (2016) 131:803–20. 10.1007/s00401-016-1545-1 27157931

[15] Paulus W. GFAP, Ki67 and IDH1: perhaps the golden triad of glioma immunohistochemistry. *Acta Neuropathol.* (2009) 118:603. 10.1007/s00401-009-0600-6 19847448

[16] Chen WJ He DS Tang RX Ren FH Chen G. Ki-67 is a Valuable prognostic factor in gliomas: evidence from a systematic review and meta-analysis. *Asian Pac J Cancer Prev.* (2015) 16:411–20. 10.7314/apjcp.2015.16.2.411 25684464

[17] Burger PC Shibata T Kleihues P. The use of the monoclonal antibody Ki-67 in the identification of proliferating cells: application to surgical neuropathology. *Am J Surg Pathol.* (1986) 10:611–7. 10.1097/00000478-198609000-00003 2428262

[18] Torp SH. Diagnostic and prognostic role of Ki67 immunostaining in human astrocytomas using four different antibodies. *Clin Neuropathol.* (2002) 21:252–7. 12489673

[19] Wang H Zhang L Zhang IY Chen X Fonseca AD Wu S S100B promotes glioma growth through chemoattraction of myeloid-derived macrophages. *Clin Cancer Res An Off J Am Assoc Cancer Res.* (2013) 19:3764–75. 10.1158/1078-0432.ccr-12-3725 23719262 PMC3725731

[20] Hessian PA Fisher L. The heterodimeric complex of MRP-8 (S100A8) and MRP-14 (S100A9). *Eur J Biochem.* (2003) 268:353–63. 10.1046/j.1432-1033.2001.01894.x 11168370

[21] Ridinger K. S100A13. Biochemical characterization and subcellular localization in different cell lines. *J Biol Chem.* (2000) 275:8686–94. 10.1074/jbc.275.12.8686 10722710

[22] Hsu K Champaiboon C Guenther BD Sorenson BS Khammanivong A Ross KF Anti-infective protective properties of S100 calgranulins. *Anti Inflamm Anti Allergy Agents Med Chem.* (2009) 8:290–305. 10.2174/187152309789838975 20523765 PMC2879674

[23] Girolamo PD. Biology of the S100 proteins–Introduction. *Micros Res Tech.* (2003) 60:537–9. 10.1002/jemt.10295

[24] Petzold A. Glial fibrillary acidic protein is a body fluid biomarker for glial pathology in human disease. *Brain Res.* (2015) 1600:17–31. 10.1016/j.brainres.2014.12.027 25543069

[25] Cotrina ML Chen M Han X Iliff J Ren Z Sun W Effects of traumatic brain injury on reactive astrogliosis and seizures in mouse models of Alexander disease. *Brain Res.* (2014) 1582:211–9. 10.1016/j.brainres.2014.07.029 25069089 PMC4164594

[26] Bodegraven EJV Asperen JVV Robe PAJ Hol EM. Importance of GFAP isoform−specific analyses in astrocytoma. *Glia.* (2019) 67:1417–33. 10.1002/glia.23594 30667110 PMC6617972

[27] Yan T Shuai-Tong Z Jing-Wei W Dong D Xiao-Chun W Guo-Qiang Y A radiomics nomogram may improve the prediction of IDH genotype for astrocytoma before surgery. *Eur Radiol.* (2019) 29:3325–37. 10.1007/s00330-019-06056-4 30972543

[28] Yiming L Zenghui Q Kaibin X Wang K Fan X Li S Radiomic features predict Ki-67 expression level and survival in lower grade gliomas. *J Neuro Oncol.* (2017) 135:317–24. 10.1007/s11060-017-2576-8 28900812

[29] Yushkevich PA Piven J Hazlett HC Smith RG Ho S Gee JC User-guided 3D active contour segmentation of anatomical structures: significantly improved efficiency and reliability. *Neuroimage.* (2006) 31:1116–28. 10.1016/j.neuroimage.2006.01.015 16545965

[30] Swami A Jain R. Scikit-learn: machine learning in python. *J Mach Learn Res.* (2013) 12:2825–30.

[31] Blagus R Lusa L. SMOTE for high-dimensional class-imbalanced data. *Bmc Bioinformatics.* (2013) 14:1–16. 10.1186/1471-2105-14-106 23522326 PMC3648438

[32] Mzoughi H Njeh I Wali A Slima MB Mahfoudhe KB. Deep multi-Scale 3D convolutional neural network (CNN) for MRI gliomas brain tumor classification. *J Digit Imaging.* (2020). 10.1007/s10278-020-00347-9 [Epub ahead of print]. 32440926 PMC7522155

[33] Ying Z Ning H Mathen P Cheng JY Krauze AV Camphausen K Automated glioma grading on conventional MRI images using deep convolutional neural networks. *Med Phys.* (2020) 47:3044–53. 10.1002/mp.14168 32277478 PMC8494136

[34] Matsui Y Maruyama T Nitta M Saito T Tsuzuki S Tamura M Prediction of lower-grade glioma molecular subtypes using deep learning. *J Neuro Oncol.* (2020) 146:321–7. 10.1007/s11060-019-03376-9 31865510

[35] Han W Qin L Bay C Chen X Yu K Miskin N Deep transfer learning and radiomics feature prediction of survival of patients with high-grade gliomas. *AJNR Am J Neuroradiol.* (2020) 41:40–8. 10.3174/ajnr.a6365 31857325 PMC6975328

[36] Bangalore Yogananda C Shah B Vejdani-Jahromi M Nalawade S Murugesan G Yu F A novel fully automated MRI-based deep-learning method for classification of IDH mutation status in brain gliomas. *Neuro Oncol.* (2020) 22:402–11. 10.1101/757385 31637430 PMC7442388

[37] Chang K Bai H Zhou H Su C Bi W Agbodza E IDHResidual convolutional neural network for the determination of status in low- and high-grade gliomas from MR imaging. *Clin Cancer Res.* (2018) 24:1073–81. 10.1158/1078-0432.CCR-17-2236 29167275 PMC6051535

